# Design of Deployable Structures by Using Bistable Compliant Mechanisms

**DOI:** 10.3390/mi13050651

**Published:** 2022-04-19

**Authors:** Tinghao Liu, Guangbo Hao

**Affiliations:** Electrical and Electronic Engineering, School of Engineering and Architecture, University College Cork, T12 K8AF Cork, Ireland; 120220250@umail.ucc.ie

**Keywords:** deployable structure, compliant mechanism, bistable mechanism, programmable behaviour

## Abstract

A deployable structure can significantly change its geometric shape by switching lattice configurations. Using compliant mechanisms as the lattice units can prevent wear and friction among multi-part mechanisms. This work presents two distinctive deployable structures based on a programmable compliant bistable lattice. Several novel parameters are introduced into the bistable mechanism to better control the behaviour of bistable mechanisms. By adjusting the defined geometry parameters, the programmable bistable lattices can be optimized for specific targets such as a larger deformation range or higher stability. The first structure is designed to perform 1D deployable movement. This structure consists of multi-series-connected bistable lattices. In order to explore the 3D bistable characteristic, a cylindrical deployable mechanism is designed based on the curved double tensural bistable lattice. The investigation of bistable lattices mainly involves four types of bistable mechanisms. These bistable mechanisms are obtained by dividing the long segment of traditional compliant bistable mechanisms into two equal parts and setting a series of angle data to them, respectively. The experiment and FEA simulation results confirm the feasibility of the compliant deployable structures.

## 1. Introduction

In recent years, deployable structures have been widely used in many fields, including but not limited to intelligent aerospace, architecture and the medical field due to the enormous dimensional deformability [1,2,3]. A novel type of closed-looped deployable structure was developed as a supporting frame for the large-diameter antenna in space engineering [4]. A negative pressure room based on deployable structures can effectively reduce the spread of the virus while ensuring the air circulation [5]. In addition, the Wren parallel mechanisms, which undergo one degree-of-freedom (DOF) Borel–Bricard motion, are designed as lattices of deployable structures as well [6].

This paragraph lists four common deployable mechanisms, which consist of different lattices [7]. The first category is scissor-like deployable structures. The deployable structures, comprised of scissor-like elements (SLEs), are widely applied because of their high loading capacity and excellent equilibrium stability [8,9,10]. The second type of deployable structure is the tensegrity structure, where tensional and compressional members co-exist. These members are connected to form a stable system [11]. In addition, another branch of deployable structures is derived from origami structures. These structures typically consist of thin membranes and slender elements [12,13,14]. In this list, the last approach to making structures deployable is to use the compliant multi-stable mechanisms as the lattices.

Compliant mechanisms can transfer or transform motion, force and energy via the deformation of flexible members [15]. Therefore, using compliant mechanisms can reduce costs and part-counts, save assembly time and also simplify manufacturing processes [16,17,18]. The wear among multi-part mechanisms can be avoided as well. Another benefit of utilizing compliant mechanisms is increasing the performance. The working precision of mechanisms can be significantly increased if replacing the traditional hinged mechanisms with compliant mechanisms [19,20,21]. The compliant mechanisms are demanded in many fields that require higher precision. A compact large-range XY compliant parallel manipulator was designed based on the characteristic of high performance [22]. In addition to the high performance, the dynamic characteristic of compliant mechanisms attracts considerable attention due to their high reliability and wide range of applications.

A compliant bistable mechanism has two stable equilibrium positions. The bistable mechanisms will tend to one of two positions if no external forces are acting on it [23]. Each stable position represents the local potential energy minimum, called the potential energy wells. A specific amount of external energy is required if the compliant bistable mechanism jumps from one stable position to another. This energy is called an energy barrier between two potential wells [24]. Additionally, there is a certain distance between two equilibrium positions. This distance can change the overall dimensions of the structure, accordingly making the structure deployable. In addition, this distance determines the deformation ability of the deployable structure.

Compliant bistable mechanisms can be categorized into two groups: spatial- and planar-compliant bistable mechanisms. The representative of the spatial bistable mechanism is the composite Laminates structure and torsion structure. The composite Laminates can change their configurations between saddle shape and cylindrical shape, thus getting its bistable characteristic [25]. Another tunable bistable component using shape memory polymers can perform twisting and rotational bistability [26]. Compared with spatial mechanisms, the planar mechanisms have the advantage of easy manufacturing and lower cost. The planar-compliant bistable mechanisms are primarily composed of straight and curved compliant segments [27,28,29]. The compliant mechanisms with straight segments have been applied extensively. A miniature latching accelerometer that does not require electrical power was designed based on compliant bistable mechanisms with straight segments [30]. When the load applied to this miniature accelerometer reaches a certain threshold, the structure will switch to a second stable state and lock. The advantage of using compliant mechanisms with straight segments is that the manufacturing process is more straightforward, and geometry parameters are easier to control. In addition to the straight segments, two curved centrally clamped parallel segments are introduced into the compliant bistable mechanisms as well [31]. This research proved the feasibility of using curved beams as segments of compliant bistable mechanisms.

Several indicators are used to measure the bistability of bistable structures, such as the critical forces and second stable position. These indicators are mainly proposed based on the force–displacement relationship. When reviewing the existed structures, it is found that these indicators of the bistable mechanism are almost impossible to adjust individually. These indicators will be influenced mutually even if changing only one geometry parameter. However, some design targets aim to optimize an individual indicator without affecting other indicators. For traditional bistable mechanisms, it is difficult to control the bistable behaviour because of the limited amount of geometry parameters. Hence, a novel analysis method is necessary. We introduced several new parameters to control the bistable behaviour using the generalized bistable model. Then, these bistable indicators can be better controlled by adjusting the geometric parameters. This work investigated how each geometry parameter influences bistable behaviour.

The aim of this work mainly involves designing deployable structures based on a programmable compliant bistable lattice with straight segments. The programmable feature refers to the highly controllable ability of bistable behaviour. The programmable feature is obtained by using the generalized bistable model. The structures are designed as both planar and spatial. Firstly, the novel type of 1D bistable lattice is proposed. The bistable lattice is tessellated into a plane to investigate the multi-stable characteristics in a single dimension. Subsequently, the lattice is curved to fit the surface of the cylinder to investigate whether the bistable characteristic will disappear when bending the lattice.

This paper is organized as below. Section 2 describes the methodologies of designing a bistable lattice with programmable behaviour. The bistable mechanism is designed from a novel perspective. The segments of the traditional compliant bistable mechanism are equally divided into two segments. The segments are defined with a series of angles. After the finite element analysis, four different types of bistable lattices are summarized. Then, the bistable lattice is optimized by revising the geometric parameters, such as the thicknesses and length of segments. The objective of optimizing the mechanism is designing a bistable lattice with larger deformation range. Section 3 mainly introduces several planar and spatial tessellations both in spatial and planar bistable lattices. Lastly, conclusions are finally drawn in Section 4.

## 2. Design of a Bistable Lattice with Programmable Behaviour

The main content of this section is to propose a method of designing a proper bistable lattice. The target bistable feature depends on the specific design aim. For instance, designing nonexplosive release mechanisms aims to make more potential energy be stored in the deformed segments [32]. Accordingly, the release mechanism can perform with higher reliability. The expected result in this work is to maximise the distance between two stable configurations while trying to increase its stability in a second stable position. Firstly, the traditional compliant bistable mechanism is introduced, and the bistable curve is also explained. Understanding the principle of the traditional bistable mechanism can contribute to the innovation of the designing method. Furthermore, four types of compliant mechanisms with compresural or tensural segments are listed by modifying geometry shapes. The double-tensural compliant mechanism is chosen to be the final mechanism. After determining the shape, the geometric parameters are analysed to optimize the bistable mechanism. Finally, the planar bistable lattice is designed and prototyped. In the experimental result, the force–displacement relationship proves that the planar compliant mechanism has the bistable feature.

**Figure 1:**
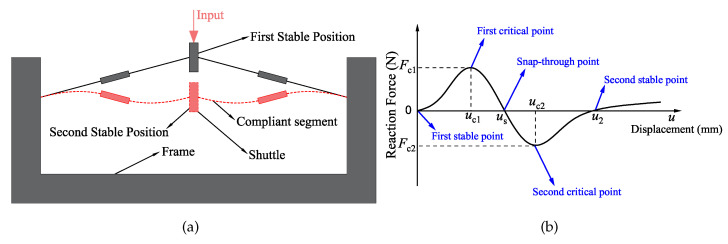
Traditional compliant bistable mechanism: (**a**) the design; (**b**) the force–displacement curve.

The traditional compliant bistable mechanism can be seen in Figure 1a. Two compliant segments under the constraint of the frame are connected to the centre shuttle. When applying a displacement input to the shuttle, the segments will bend. Due to the symmetry characteristic, the shuttle will move along the centre line, which is just like moving in a guide. The bistable curve, which is shown in Figure 1b, can represent the typical force–displacement relationship of the bistable mechanism. The displacement *u* is defined as the distance that the shuttle has moved along its centre axis. The positive direction of displacement *u* is the same as the input direction. The reaction force *F* represents the reaction force of the shuttle along the centre axis. Some points in the bistable curve are defined to explain their significance. The diagram starts from the original point, i.e., the first stable point. No external force or displacement is input to the system at that status. Therefore, the structure is in stable status at that position. When the displacement increases, the reaction force will become larger. This process lasts until the first extreme point, called the first critical point. At that point, the displacement and reaction force are defined as $u_{c1}$ and $F_{c1}$, respectively. If we continue increasing the displacement *u*, the bistable mechanism will arrive at its snap-through point $u_{s}$. When the displacement of the shuttle reaches the snap-through position, the shuttle starts to act opposite to the first stable point and quickly shifts to the second stable configuration. The displacement of the second critical point is defined as $u_{c2}$. Correspondingly, the reaction force at the second critical point is $F_{c2}$. If the second critical force $F_{c2}$ is above zero, the structure has only one equilibrium position. The second stable position is located at $u_{2}$. The structure can keep its configuration at that position without adding any external force. The parametric analysis is mainly based on the above-defined parameters since they represent the performance of bistable mechanisms.

**Figure 2:**
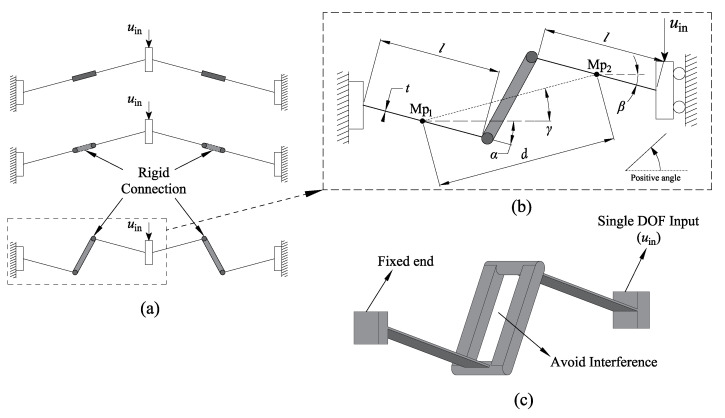
The generalized compliant bistable mechanism: (**a**) the derivation process from traditional compliant bistable mechanism to generalized mechanism; (**b**) The defined parameters; (**c**) The multi-layer analysis model.

A novel method of designing a compliant bistable lattice is proposed in this work. The schematic of the generalized compliant bistable mechanism can be seen in Figure 2. The traditional compliant segments are divided into two equal parts. These two parts are fixed by a rigid connection. The aim is to investigate whether the segments will perform bistable behaviour when changing the angle of each half-segment. In this method, the middle point of each half-segment is fixed with a relative location. The relative position of the two middle points (Mp${}_{1}$ and Mp${}_{2}$) is defined by two parameters, i.e., γ and *d*. The half-segments are set in a series of angles. The angle range varies from 0${}^{\circ }$ to 360${}^{\circ }$ to make an investigation with relatively more extensive coverage. The angle intervals are initially set as 60${}^{\circ }$ to explore the existence of bistable features. These structures are designed in three layers. The middle layer is the compliant half-segments. The problem of interference, when changing the angles of half-segments, is solved by adding out-plane rigid connections. These connections are designed with enough rigidity to resist the inner force. In addition, the deformation of these connections is small enough to be ignored.

**Figure 3:**
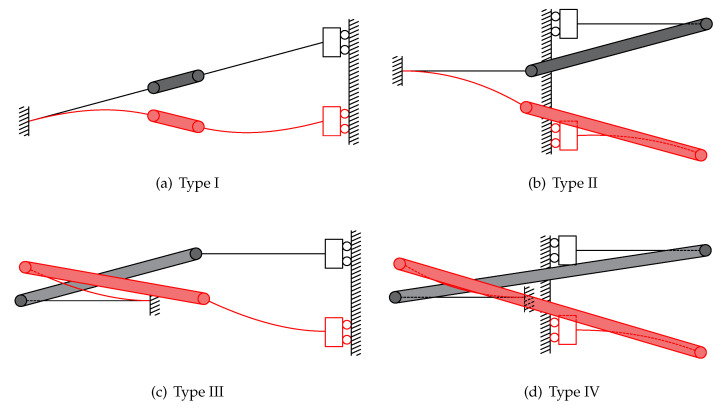
Compliant bistable mechanisms derived from the generalized model when the parameters α and β are set at different angles. These mechanisms are designed based on the multi-layer analysis model. The left half-segments are anchored to the frame, and the right half-segments are connected to the shuttle: (**a**) compliant compresural–compresural bistable mechanism (traditional compliant bistable mechanism); (**b**) compliant tensural–compresural bistable mechanism; (**c**) compliant compresural–tensural bistable mechanism; (**d**) compliant tensural–tensural bistable mechanism.

Four types of compliant bistable mechanisms are derived from the above generalized model. They can be obtained from Figure 3. Each of them consists of compresural or tensural half-segments. The compresural segment refers to the compliant part that will be under compressive stress during the flexure. The naming of tensural segments is based on the same principle as compresural segments [33,34]. Type one is the traditional compliant bistable mechanism. Both of the two half-segments are under compresural stress when displacement input exists. The second type of bistable mechanism has both compresural and tensural half-segments. The compresural half-segment, which is fixed to the frame, is placed on the left-hand side. The tensural half-segment is connected to the shuttle. The shuttle is represented by a roller on a guide in the schematic diagram due to its symmetric feature. After that, another bistable mechanism, whose compresural half-segment is fixed to the frame, is proposed. The tensural half-segment of this mechanism is connected to the shuttle. The mechanical properties of a compliant compresural–tensural bistable mechanism have been investigated by previous researchers [34]. The last type of compliant bistable mechanism is designed with a double tensural half-segment. The angle parameters of these compliant bistable mechanisms are shown in Table 1. This work mainly focuses on the bistable mechanisms with double tensural segments.

Finite element methods are used to analyse static or dynamic objects and systems. This method decomposes an object or system into a geometric model composed of multiple interconnected, simple, independent points. Linear static analysis is one type of method in which the displacement and force applied to the structure have a linear relationship. The stress is regarded as still in the elastic range during the linear analysis process, and its stiffness matrix is constant. However, the linear static analysis is insufficient for the mechanism with large deformation since it cannot reflect the actual relationships between force and displacement. Hence, in this paper, nonlinear FEA is used to analyse the bistable behaviour of lattices. The software used for FEA analysis in this paper is Strand7. During the simulation, a square mesh is applied to subdivide the structure. The maximum edge length of each mesh is set as 3% of the overall geometry size. Both geometry and material nonlinearity are considered. The Strand7 nonlinear static solver uses an algorithm based on the generalized Newton–Raphson method. This algorithm has the following features:

- The load increments can be defined easily.
- Multiple freedom cases can be used.
- The stiffness matrix need not be updated in every iteration.
- Restart can be used to continue a previously completed or aborted solution.

**Figure 4:**
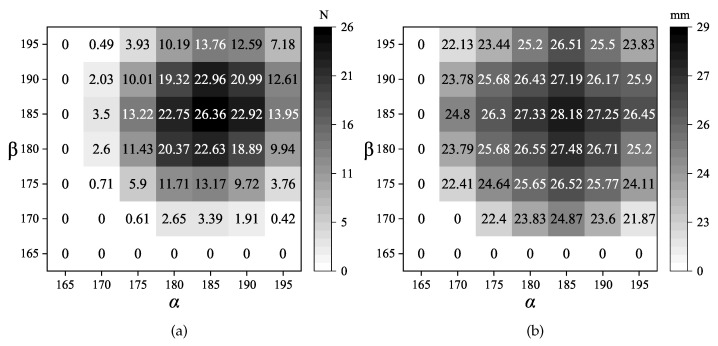
The switch-back forces and second stable positions when α and β are close to 180${}^{\circ }$. When the switch-back force is 0, it represents the structure is absent of bistable characteristics: (**a**) the forces at the second critical points; (**b**) the displacements at the second stable points.

The two interrelated parameters α and β are both 180${}^{\circ }$ in the preliminary analysis process. In order to perform the analysis in higher resolution, the angle interval is decreased to 5${}^{\circ }$. The range of angles varies from 165${}^{\circ }$ to 195${}^{\circ }$. The reaction forces at the second critical point and the displacement of the second stable points are listed in Figure 4. In Figure 4a, these values represent the magnitudes of the forces at the second critical point. The maximum force located at the point where α = β = 185${}^{\circ }$. It is found that in Figure 4b, the maximum displacement of the second stable status also happens in that angle pair. Hence, the angles α and β are decided to be 185${}^{\circ }$ to get a larger deformation range and higher stability in the second stable status. A larger force at the second critical point means the mechanism is more stable in the second equilibrium position. Increasing the stroke of the shuttle of the deployable lattice can expand the range of deformation. Hence, these two parameters are the key points of designing a bistable lattice. After the determination of α and β, the next step of designing is to determine the other four parameters: angle γ, distance *d*, segment length *l* and segment thickness *t*.

**Figure 5:**
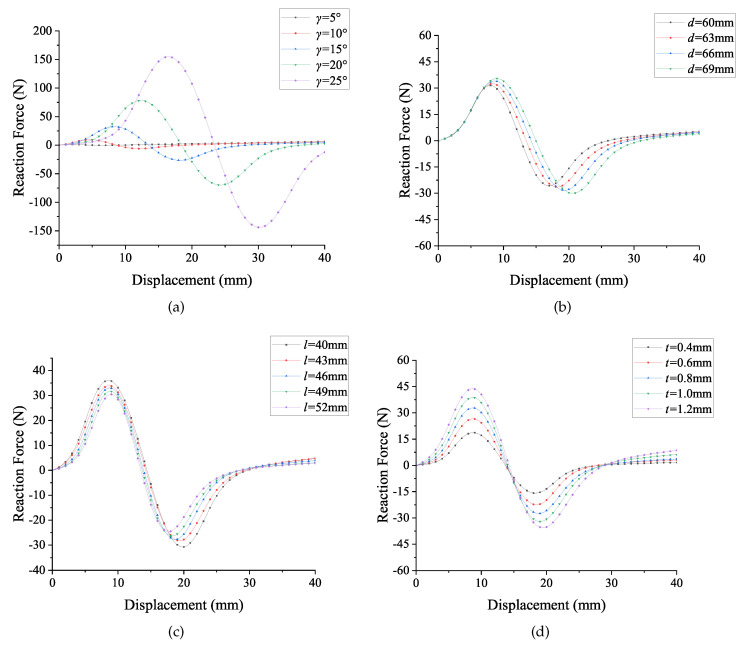
The bistable curves under different variables: (**a**) the angle γ as variable; (**b**) the distance between middle points *d* as variable; (**c**) the segment length *l* as variable; (**d**) the thickness of segments *t* as variable.

The first parameter that needs to be investigated is the angle γ, which is defined between the line connecting two middle points and the horizontal line. The γ is set in a series of angles, varying from 5${}^{\circ }$ to 25${}^{\circ }$. As can be seen in Figure 5a, the forces at the first critical point $F_{c1}$ of the bistable mechanism increase with the increment of angle γ. For example, when the angle is set as 25${}^{\circ }$, the force $F_{c1}$ is around 150 N. The magnitude of the force $F_{c1}$ represents the resistance of the changing configuration. In addition, the distance between two configurations ($u_{2}$) will be larger when increasing the angle γ. However, if the switch force is too big, it will be difficult for the mechanism to change its configuration. In terms of designing the deployable structure in this work, the relatively large forces at critical points are not necessary. Hence, the angle γ is finally decided to be 25${}^{\circ }$.

The following parameter that needs to be determined in the design process is the distance *d* between two middle points. The FEA result is shown in Figure 5b, the distance is set in four variables that vary from 60 mm to 69 mm. The change of *d* causes a tiny influence on the magnitude of the forces at the critical points. When the distance *d* becomes larger, the magnitude of the force $F_{c2}$ will increase. Moreover, the position of these two critical points, $u_{c1}$ and $u_{c2}$, are influenced as well. The position $u_{c2}$ will tend to increase when setting a longer point distance. The switch position $u_{c1}$ will increase as well, although the magnitude of change is not as large as $u_{c2}$. If the distance *d* is designed to be too large, it will affect the overall size of the compliant bistable lattice. The overall size of the bistable lattice is limited to the manufacturing feasibility. In contrast, the distance *d* has little effect on the bistable behaviour. Hence, the distance *d* is determined as 63mm, which is a moderate value in the range.

Conversely, in terms of the segment length *l*, the reaction forces at the critical points will become smaller when increasing the length. The maximum switch force in Figure 5c occurs when setting 40 mm as the segment length, which is the shortest segment in that range. The position of the second equilibrium point $u_{2}$ keeps stable when changing the lengths of segments. Changing the segment length has almost no influence on the equilibrium positions. The valid range of the segment lengths is limited by the geometric shape. The segment length is determined as 46 mm since the reaction forces are moderate. In addition to the segment length, if the thickness of segments *t* is set as the variable during the analysis process, the variation of thickness will only affect the magnitude of forces. The position of the critical points will not change as well. Another consideration is that the thickness of segments (*t*) is limited to the manufacturing precision. A very thin segment is difficult to manufacture, especially for CNC manufacturing methods, since the precision of thin membranes will be influenced by the milling or cutting stress. The thickness of the segments *t* highly depends on the manufacturing quality. Therefore, increasing the thickness of segments contributes to easier manufacturing. The thicknesses of segments are determined as 1.2 mm to maximise the feasibility of prototyping.

In summary, when changing the previous defined geometry parameters, the bistable curve will be affected to some extent. The adjustment of the bistable curve is mainly based on changing these parameters. And the design purpose determines which specific geometry should be adjusted. The adjustment method depends on the particular demand of adjustment. If the only need is changing the magnitude of forces, the thickness of segments (*t*) might be the only parameter that needs to be adjusted. If both the second stable positions and force magnitudes need to be adjusted, changing the angle (γ) is a good solution.

Through the above process of adjusting the geometry parameters, the final planar-compliant bistable lattice is determined. The parameters that are used in the planar mechanism can be found in Table 2. The rigid connections in the multi-layer analysis model are replaced by another type of connection. The bistable lattice is designed in plane.

**Figure 6:**
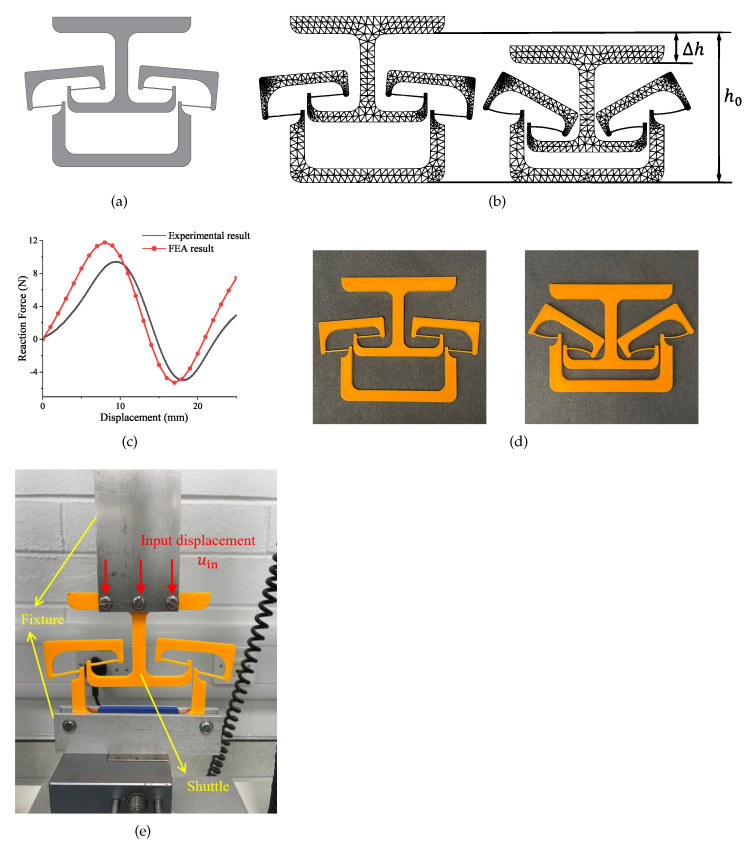
The planar compliant bistable mechanism: (**a**) the design of planar bistable mechanism; (**b**) the deformation result of FEA simulation; (**c**) the comparison between FEA simulation and experiment; (**d**) the 3D-printed prototype using PLA as material; (**e**) the experimental setup.

The planar bistable lattice is shown in Figure 6a. The final determined lattice is simulated by using FEA software. The mesh result and deformation result is shown in Figure 6b. A triangular mesh is selected since it is better fit to the curve profiles. In this design, the $h_{0}$ is designed as 210 mm, the shrunken length of the lattice Δh is 42.61 mm. The lattice is prototyped in half scale due to the limitations of the 3D printer. The 3D printer used is an Ultimaker S5. The build volume of this 3D printer is 330× 240 × 300 mm. The 3D printing software used to slice the model is Ultimaker Cura. During the prototyping process, the print core used in the printer is AA 0.4. The infill of the prototype is set as 100% to achieve the best performance. The material used for prototyping is Polylactide (PLA). The material properties are shown in Table 3. The centre shuttle of the 3D manufactured model can be pushed to its second stable configuration. The two distinct configurations can be seen in Figure 6d.

A compress experiment is performed in this work. The comparison between the experimental result and the FEA simulation result is shown in Figure 6c. The setup of the experiment is shown in Figure 6e. The bistable lattice is fixed by two fixtures on the above and bottom, respectively. The shuttle moves along the direction of input displacement. This experiment was performed on the texture analyser (TA. Hd plus texture static test system), which is a load–displacement measurement system that moves in one single direction to compress or stretch the mechanism. A load cell of 5 (kg) was selected with a force resolution of 0.1 (g). The displacement was applied as the input to the system. The upper fixture moved downward at a shallow speed (0.1mm/s) to minimize the dynamic effect. Due to the limitation of devices, the only experiment performed in this work was investigating the bistable behaviour of planar mechanism. As can be seen in the comparison, some error exists. The error might be caused by the following reasons:

- The orthotropic functional properties of additively manufacturing.
- The uneven thickness of the segments.
- The frame lacks rigidity.
- The plastic deformation during the deformation process.

**Figure 7:**
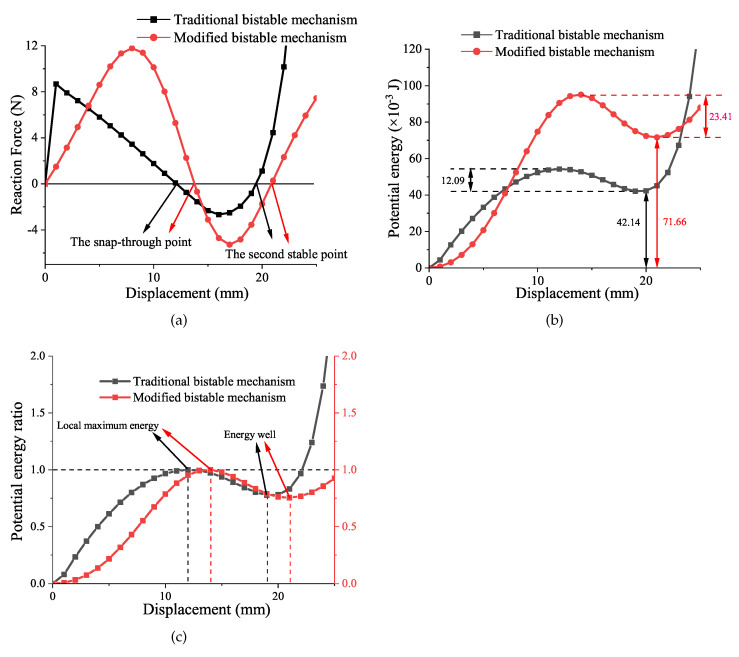
The comparison between the generalized bsitable mechanism and the traditional bistable mechanism: (**a**) the relationship between reaction forces and displacement; (**b**) the potential energy of mechanisms; (**c**) the ratio of potential energy compared to its local maximum energy.

A comparison between the generalized planar-compliant bistable mechanism with double tensural segments and the traditional bistable mechanism is performed in this work. The comparison results are shown in Figure 7. These two mechanisms are designed with the same parameters except the angle α and β. As can be seen in Figure 7a, the generalized mechanism has the benefit of a relatively larger distance between two stable configurations. By utilizing the generalized mechanism as the lattice of deployable structure, the structure can increase its deployment range. In addition, the critical point forces $F_{c1}$ and $F_{c2}$ of the generalized mechanism are larger. This advantage can help to increase the stability of the deployment. When designing a deployable structure, consideration must be given to the ability of the structure to resist shock and load. The harder the switching process performs, the more energy it takes for the lattice to change its configuration. In other words, the structure has a higher ability to resist shock and load in its first and second stable state. Therefore, the larger these forces are, the more stable the deployable structure is. In Figure 7b, the results show that the generalized mechanism stored more potential energy in its second stable status compared to the traditional bistable mechanism. This benefit can increase the efficiency of energy conversion and harvest. In addition, a ratio of potential energy is introduced to the analysis process which is shown in Figure 7c. This ratio is defined as the result of the magnitudes of potential energy divided by the local maximum energy during the deformation process. As mentioned previously, the energy well is located at the position of the second stable point. At this position, the potential energy arrives at the position of local minimum potential energy. Therefore, in the position of the energy well, if there is no external force adding to the mechanism, the structure will keep in its stable status. The advances of the generalized mechanism are summarized as follows:

- Larger deformation range between two stable configurations.
- Better stability before/after deployment.
- More potential energy stored in the second stable status.

## 3. Tessellated Deployable Structure

In the designing process of a structure with multi lattices, the tessellation method has various types. This section mainly discusses two modes of tessellating the bistable lattices into 1D and cylindrical configurations. In the 1D configuration, the bistable lattice is series connected to derive a multistable deployable structure. In the cylindrical configuration, the lattices are bent to form a cylindrical structure to investigate whether the bistable characteristic will disappear when using the curved bistable lattice.

### 3.1. 1D Deployable Structure

**Figure 8:**
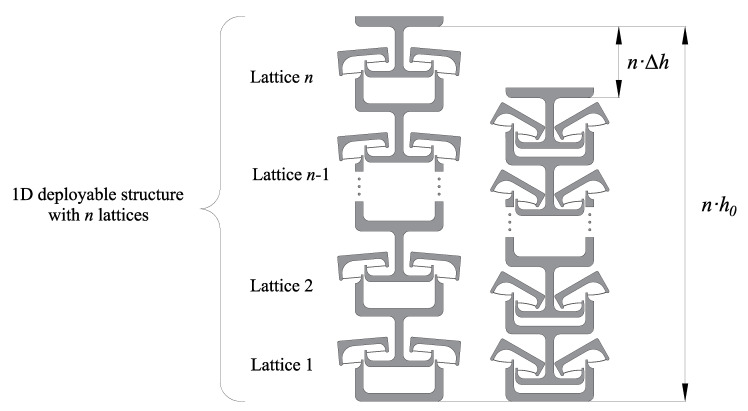
The multi-stable mechanism derived by tessellating bistable lattices.

A multi-stable mechanism can be derived from tessellating the bistable lattices. In this part, the lattice is designed to be fitted into a single dimension structure. As mentioned previously, the shuttle of the bistable lattice moves along its centre axis. A theoretical multi-stable 1D deployable structure is obtained by overlapping the bistable lattice centre line. The series-connected mechanism can be seen in Figure 8. As can be seen, there are *n* lattices series-connected to form an entire 1D deployable structure. The overall height of the mechanism before deployment is $n\cdot h_{0}$. The deformation range of this 1D structure is n·Δh.

In this work, the key content of the investigation is the three-connected multi-stable mechanism. This is because three lattices connected can perform a more generalized deformation compared to the two-connected structures. Each lattice of the deployable structure has two distinct stable configurations. The first stable position of them is when there is no deformation.

**Figure 9:**
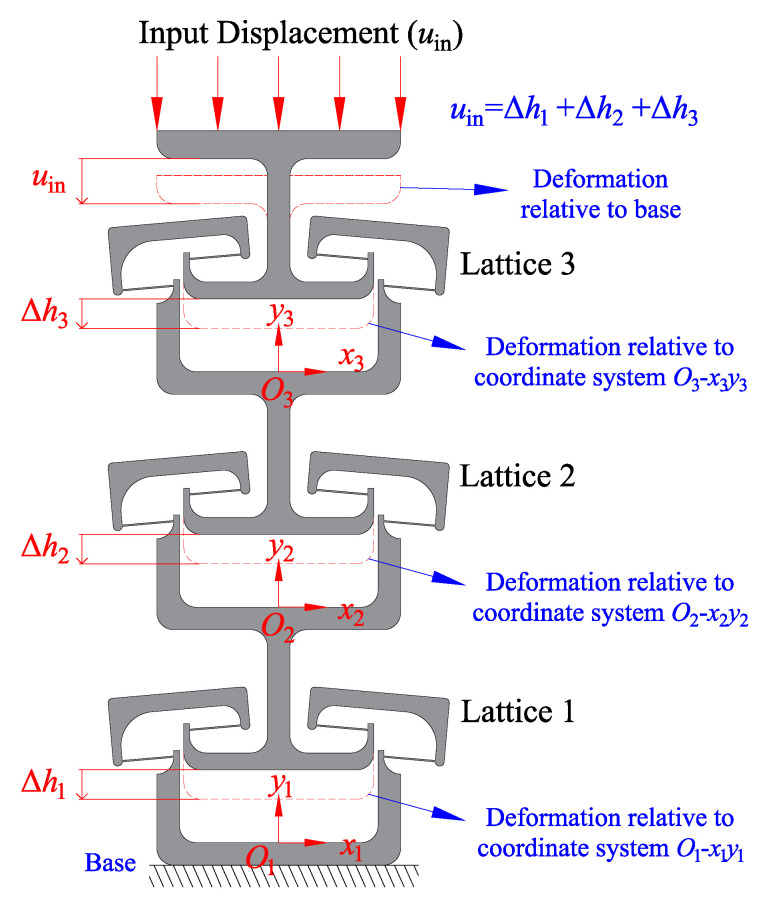
The parameters defined to describe the process of deformation.

To better explain the process of the deformation, several parameters are introduced to the three-connected structure. The definition of these parameters can be seen in Figure 9. The FEA simulation operates based on a constant input displacement on the top of lattice three. The input displacement is named as $u_{in}$. In this mode, the displacement $u_{in}$ increases linearly. The deformation $u_{in}$ is relative to the base which is fixed. To better explore the deformation process of each lattice, the deformation distance $\Delta h_{n}$ is introduced to the model (n is the order of lattices). The deformation distance $\Delta h_{n}$ is relative to the corresponding coordinate system $O_{n}-x_{n}y_{n}$. The coordinate system is fixed on the frame centre of each lattice. In addition, the magnitude of $u_{in}$ is equal to the sum of $\Delta h_{n}$.

The determination of multi-stable characteristics is based on measuring the relative displacement between frame and centre shuttle. When the structure is under load, the bistable lattice will change its configuration when the displacement of the shuttle reach and pass the snap-through point. It means that when the displacement *u* reaches the snap-through position $u_{s}$, the bistable lattice will change its configuration to the second stable equilibrium position. In addition, these lattices are series-connected but do not change their configuration at the same time. The order of change depends on the specific load situation.

As can be seen from the single bistable curve in Figure 1b, when the input displacement is before the snap-through point, the elastic deformation of the structure produces a force opposite the direction of motion. As the displacement increases, the reactive force of the structure tends to bring it back to its initial position. However, after passing the snap-through point, the reaction force of the structure becomes negative. The force after this point promotes the structure to switch to the next stable state. Without constraints, this reactive force would cause the displacement of shuttles to abruptly switch to a second stable state. For multiple series-connected bistable structures, when only the uppermost end is loaded with an overall input displacement, the relative position between the shuttle and the frame is not constrained. When a bistable structure switches stable configurations, its shuttle must pass through its snap-through position. The most intuitive phenomenon of the switching configuration of a bistable lattice is that there is a mutation in displacement and will not return to the first stable state. Therefore, we judge the sequence of lattice switching configuration in the structure by observing the sequence of generating displacement mutation. If three springs are connected in series, and the same load is applied to them, the springs will deform uniformly and linearly because the stiffness of the springs is constant. However, for the series-connected multistable mechanisms proposed in this paper, their nonlinear behaviour in the force and displacement relationship results in them not moving uniformly like the springs. When the overall displacement u${}_{in}$ is input, each lattice does not produce a uniform displacement because the force between them makes it difficult to achieve equilibrium.

**Figure 10:**
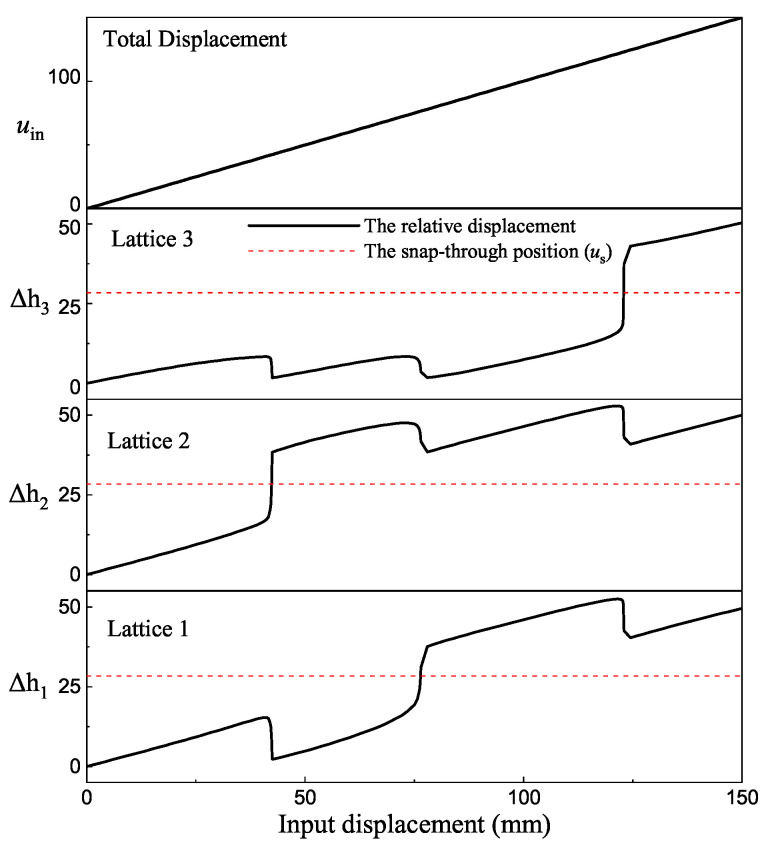
The displacement of each element under the load of input displacement.

In Figure 10, the overall displacement of the three-connected structure is set as increasing at a constant rate. The sum displacement of these three lattices is equal to the overall displacement. As mentioned, when the displacement of each shuttle is larger than $u_{s}$, the lattice will change to its second stable configuration. Due to the restraint of displacement, when a lattice changes its configuration, the other two lattices will also be influenced. The figure marks the snap-through position as a red dashed line. When the displacement $\Delta h_{n}$ is larger than the switch position, the lattice will change its configuration to the second stable status. Based on the FEA simulation result, the first lattice that changes to the second stable status is Lattice 2. From the figure, the first intersection between lines happened in the field of Lattice 2. The second lattice that changes its configuration is Lattice 1. The last one is the Lattice 3. The displacement result shows that three lattices change their configuration in total. Therefore, the result proves that the structure in this situation has four stable equilibrium status. The above diagram proves that the structure with three series-connected bistable lattices has four distinct equilibrium positions. When the displacement is set as input, lattices will affect each other during the configuration change process.

### 3.2. Cylindrical Deployable Structure

Designing the cylindrical structure provides a possible solution to the cylindrical bistable mechanism with programmable behaviour. Compared with the cylindrical bistable mechanism composed of traditional bistable lattice, this generalized mechanism can better be targeted optimized due to several more geometric parameters being introduced. Hence, the method of optimizing the bistable behaviour can be applied to the cylindrical structure as well. Therefore, the proposal of cylindrical structure is not only designing a bistable structure but also introducing the programmable characteristic into it. This part proposes a cylindrical deployable structure based on the curved programmable bistable lattice. If the cylindrical deployable lattice is designed in a multi-layer structure, it will become a long telescopic tube with no diameter change during the deformation. This mechanism can be used as an engagement aid for transport pipelines.

**Figure 11:**
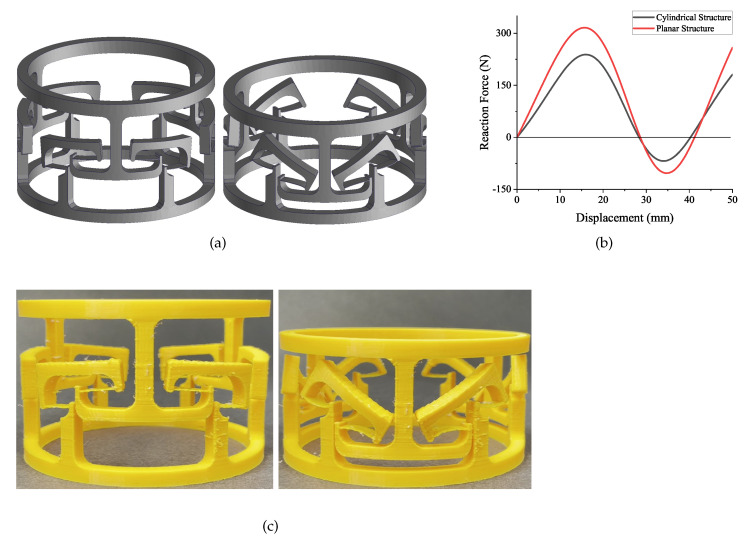
The cylindrical deployable structure: (**a**) the design; (**b**) the FEA simulation result; (**c**) two stable configurations of the cylindrical prototype.

The structure and its second stable configuration can be seen in Figure 11a. The cylindrical structure is prototyped by 3D printing technology as well. Then, an FEA simulation was performed. The result of the force–displacement relationship is shown in Figure 11b. This curve has three intersection points with the horizon line. This curve meets all the features of the bistable curve. Hence, the cylindrical structure has bistable characteristics. In addition, the 3D model can be found in Figure 11c. The 3D model proves that the structure has two stable configurations, i.e., it will keep in two stable statuses when there is no force acting on it. Therefore, this result proves that the bistable characteristic will not disappear when bending the lattices into a cylinder shape.

A comparison between planar and cylindrical structures was performed. Both of these two structures consist of three bistable lattices. The lattices are arranged horizontally. The result illustrates that the cylindrical structure has relatively lower critical forces. This might be caused by the out-of-cylinder motion during the deformation process. Furthermore, the cylindrical structure design demonstrates the feasibility of translating the concept into a potential practical application. The aim of converting the flat 2D model to a 3D cylindrical model is to investigate the bistable behaviour in a higher dimension.

## 4. Conclusions

In this work, we presented two types of deployable structures including a 1D and a cylindrical model. These structures are designed based on the programmable bistable lattices. We performed a series of FEA simulations and parametric analysis to obtain a proper bistable lattice. Four types of bistable lattices derived from traditional compliant bistable mechanisms are listed in this work as well. The experimental results and FEA simulations prove that the designed structure has more than one stable status when no external force is acting on it. The breakthrough in this work provides more options for designing the deployable structure and bistable mechanisms.

## Figures and Tables

**Table 1:** The angle parameters of compliant bistable mechanisms.

	Type I	Type II	Type III	Type IV
α	15${}^{\circ }$	0${}^{\circ }$	180${}^{\circ }$	180${}^{\circ }$
β	15${}^{\circ }$	180${}^{\circ }$	0${}^{\circ }$	180${}^{\circ }$
γ	15${}^{\circ }$	15${}^{\circ }$	15${}^{\circ }$	15${}^{\circ }$

**Table 2:** The determined geometry parameters of the planar bistable lattice.

α	β	γ	*d*	*l*	*t*
185${}^{\circ }$	185${}^{\circ }$	25${}^{\circ }$	63 mm	46 mm	1.2 mm

**Table 3:** The mechanical properties of Polylactide (PLA).

Property	Tensile Modulus	Flexural Moudulus	Yield Tensile Stress	Yield Elongation
**Value**	2346.5 MPa	3150.0 MPa	49.5 MPa	3.3%
